# Unlocking the Potential of RNA Sequencing in COVID-19: Toward Accurate Diagnosis and Personalized Medicine

**DOI:** 10.3390/diagnostics15020229

**Published:** 2025-01-20

**Authors:** Heba M. Saad Eldien, Abdulrahman H. Almaeen, Ahmed Abo El Fath, Ahmed E. Taha, Rehab Ahmed, Hassabelrasoul Elfadil, Helal F. Hetta

**Affiliations:** 1 Department of Anatomy, College of Medicine, Jouf University, Sakaka 72388, Saudi Arabia; 2 Department of Pathology, College of Medicine, Jouf University, Sakaka 72388, Saudi Arabia; ahalmaeen@ju.edu.sa; 3 Tropical Medicine and Gastroenterology Department, Assiut University Hospital, Assiut 71515, Egypt; ahmed111@aun.edu.eg; 4 Microbiology and Immunology Unit, Department of Pathology, College of Medicine, Jouf University, Sakaka 72388, Saudi Arabia; aeattia@ju.edu.sa; 5 Department of Medical Microbiology and Immunology, Faculty of Medicine, Mansoura University, Mansoura 35516, Egypt; 6 Division of Microbiology, Immunology and Biotechnology, Department of Natural Products and Alternative Medicine, Faculty of Pharmacy, University of Tabuk, Tabuk 71491, Saudi Arabia; rahmed@ut.edu.sa (R.A.); habdelgadir@ut.edu.sa (H.E.)

**Keywords:** COVID-19, RNAseq, transcriptome, host responses, gene, precision medicine, prognosis

## Abstract

COVID-19 has caused widespread morbidity and mortality, with its effects extending to multiple organ systems. Despite known risk factors for severe disease, including advanced age and underlying comorbidities, patient outcomes can vary significantly. This variability complicates efforts to predict disease progression and tailor treatment strategies. While diagnostic and therapeutic approaches are still under debate, RNA sequencing (RNAseq) has emerged as a promising tool to provide deeper insights into the pathophysiology of COVID-19 and guide personalized treatment. A comprehensive literature review was conducted using PubMed, Scopus, Web of Science, and Google Scholar. We employed Medical Subject Headings (MeSH) terms and relevant keywords to identify studies that explored the role of RNAseq in COVID-19 diagnostics, prognostics, and therapeutics. RNAseq has proven instrumental in identifying molecular biomarkers associated with disease severity in patients with COVID-19. It allows for the differentiation between asymptomatic and symptomatic individuals and sheds light on the immune response mechanisms that contribute to disease progression. In critically ill patients, RNAseq has been crucial for identifying key genes that may predict patient outcomes, guiding therapeutic decisions, and assessing the long-term effects of the virus. Additionally, RNAseq has helped in understanding the persistence of viral RNA after recovery, offering new insights into the management of post-acute sequelae, including long COVID. RNA sequencing significantly improves COVID-19 management, particularly for critically ill patients, by enhancing diagnostic accuracy, personalizing treatment, and predicting therapeutic responses. It refines patient stratification, improving outcomes, and holds promise for targeted interventions in both acute and long COVID.

## 1. Introduction

Infectious diseases, particularly viral epidemics, have consistently posed significant threats to global health, as demonstrated by the persistent COVID-19 pandemic. Despite advancements in drug and vaccine development, the emergence of novel viruses, such as SARS-CoV-2, underscores the vulnerability of populations worldwide and highlights the urgent need for rapid and effective diagnostic and therapeutic strategies [1,2,3,4,5].

**Figure 1:**
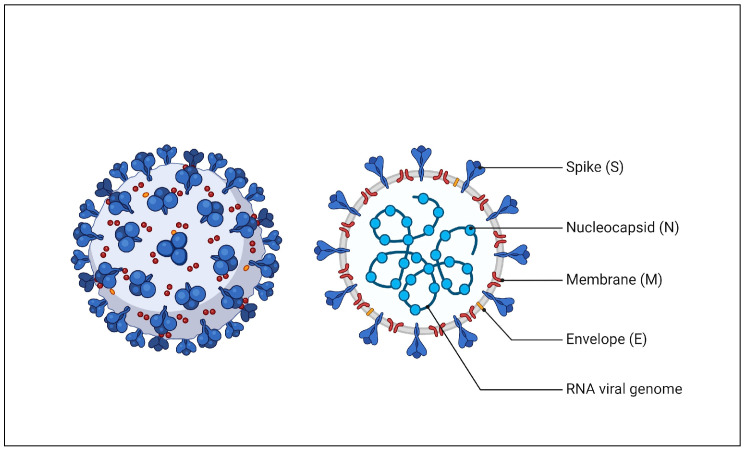
Structure of human coronavirus.

Coronaviruses (CoV) are members of the subfamily Coronavirinae within the family Coronaviridae (order Nidovirales). They are structured from an envelope and a single-stranded, positive-sense RNA molecule with approximately 30,000 nucleotides, measuring between 60 and 140 nm in diameter and covered in spike-like projections that, when viewed under an electron microscope, resemble a crown [6,7,8] (Figure 1). Coronaviruses (CoVs) are classified into four types: alpha-, beta-, gamma-, and delta-CoVs. Among these, human coronaviruses (HCoVs) 229E and NL63 belong to alpha-CoVs, while beta-CoVs include HCoV-HKU1, HCoV-OC43, SARS-CoV-1, MERS-CoV, and SARS-CoV-2 [9,10,11,12,13]. The spike proteins of CoVs play a crucial role in determining the virus’s antigenicity and pathogenicity, as they are key to its biological and infectious characteristics [14,15].

HCoV-229E, HCoV-NL63, HCoV-HKU1, and HCoV-OC43 are endemic human coronaviruses responsible for 15–30% of common cold cases in adults, typically causing mild to moderate upper respiratory infections. However, in vulnerable groups like the elderly, children, and immunocompromised individuals, these viruses can lead to severe conditions such as bronchiolitis, pneumonia, and croup [16,17,18,19,20,21,22]. By contrast, beta-coronaviruses such as MERS-CoV, SARS-CoV-1, and SARS-CoV-2 are associated with severe disease and high mortality rates [18,23,24,25]. In late 2019, a pneumonia outbreak with an unexplained cause appeared in Wuhan, China. Despite containment and quarantine efforts, COVID-19 cases rapidly surged [26]. By August 2024, the World Health Organization reported over 776 million confirmed SARS-CoV-2 cases globally [27].

SARS-CoV-2, the seventh known human coronavirus, was found to share 89% genetic similarity with bat SARS-CoVZXC21 and 82% with human SARS-CoV. Research revealed that its spike (S) protein has a strong affinity for the ACE2 receptor, enabling efficient viral entry into human cells and driving its high infectivity [28,29,30].

COVID-19 has brought unprecedented global health challenges, revealing insights into viral pathogenesis, immune responses, and the dynamics of viral mutations during transmission and spread. These mutations can alter the virus’s transmissibility, virulence, and impact on disease severity, contributing to the emergence of new variants [31,32,33,34,35,36].

Non-coding RNAs (ncRNAs), such as long non-coding RNAs (lncRNAs) and microRNAs (miRNAs), play crucial roles in regulating gene expression and cellular processes. Unlike mRNAs, which code for proteins, ncRNAs influence various biological processes through their effects on transcription, translation, and post-transcriptional modifications [37]. In the context of viral infections like COVID-19, ncRNAs are crucial in modulating immune responses, inflammation, and viral replication. The SARS-CoV-2 virus, responsible for COVID-19, interacts with the host’s ncRNA machinery to alter immune responses and contribute to disease severity, with outcomes ranging from asymptomatic infections to severe acute respiratory distress syndrome (ARDS) and death [38,39].

RNA sequencing (RNAseq) has proven valuable in identifying altered expression of ncRNAs [38,39,40,41] and other transcriptomic components in patients with COVID-19 [42,43,44]. This technology offers significant potential for early diagnosis, severity prediction, and personalized therapeutic strategies by analyzing the molecular mechanisms underlying the disease. Through RNAseq, we gain a deeper understanding of host–pathogen interactions, offering new opportunities for managing COVID-19.

## 2. Methodology

Inclusion Criteria: Studies that focus on the application of RNA sequencing in COVID-19 research, specifically those addressing host immune responses, viral dynamics, and biomarkers of disease progression.

Exclusion Criteria: Studies that do not use RNA sequencing as the primary method, those in non-English languages, and non-peer-reviewed articles.

We conducted a comprehensive literature review by searching the following databases: PubMed, Web of Science, and Scopus. The search strategy employed a combination of Medical Subject Headings (MeSH) terms and targeted keywords to ensure comprehensive coverage of the topic. The following keywords were utilized ‘RNA sequencing’, ‘COVID-19’, ‘transcriptomics’, ‘biomarkers’, and ‘viral-host interactions.

## 3. Benefits of RNAseq in the Diagnosis of Blood Transcriptome Changes in Patients with COVID-19

RNAseq offers significant potential in diagnosing blood transcriptome changes in patients with COVID-19 [45,46]. Despite its high cost, RNAseq provides critical insights into gene expression changes in host tissues and cells following SARS-CoV-2 infection, helping to unravel disease mechanisms and host-pathogen interactions [44,47,48]. Transcriptomic analyses of blood samples from patients with COVID-19 have identified differentially expressed genes linked to immune and inflammatory responses, as well as regulatory networks [49,50]. These findings can aid in discovering diagnostic and prognostic biomarkers and identifying novel therapeutic targets for personalized treatment as shown in Table 1 [51,52,53,54].

For example, severe cases of COVID-19 are characterized by a significant increase in neutrophil-associated transcripts, including *DEFA1*, which can rise by as much as 25-fold, coupled with a marked decrease in T cell-associated transcripts, such as those related to the T cell receptor, which are reduced by 3 to 5 times [42]. Wargodsky et al. revealed a link between heightened neutrophil activity, diminished T cell function, and increased severity of COVID-19. They identified blood DEFA1 RNA levels and neutrophil elastase activity—key components of neutrophil extracellular traps (NETs)—as potential biomarkers reflecting host immune responses following viral infection. The authors concluded that neutrophil elastase might be useful as a rapid point-of-care assay for the detection of neutrophil activation [42]. However, the study noted challenges in measuring RNA biomarkers in clinical settings due to the instability of RNA in the blood and the complexity of RNA purification and quantification. Additionally, transcriptomic studies on lung and bronchoalveolar lavage fluid samples have highlighted significant B cell activation during SARS-CoV-2 infection, further supporting the utility of RNAseq in understanding COVID-19 immunology [55].

## 4. RNAseq Analysis in Differentiation Between Different Disease Courses

Faridl et al. compared RNAseq profiling in nasopharyngeal swabs from asymptomatic and slightly symptomatic patients with COVID-19 [56]. Nasopharyngeal swabs from patients with COVID-19, particularly those in the asymptomatic group, showed altered cilia linked to altered cell cycle, macroautophagy, and epigenetics. By contrast, patients with mild symptoms showed overexpression of autophagy-regulating genes as well as alteration in RNA, leading to the dysregulation of RNA processing and translation with subsequent severe outcomes. Asymptomatic cases exhibited mRNA splicing, while mildly symptomatic patients exhibited modified RNA transport. Meanwhile, severe symptomatic instances could be caused by a large disruption in RNA translation, and there was no link between different virus strains and the severity of the disease [56].

Bass et al. conducted a comparative analysis between mild, moderate, and severe patients with COVID-19 through the analysis of both multiple sc-RNAseq and bulk RNAseq data from different organ samples [57]. The authors discovered an increase in innate immune activity, perhaps due to overexpression of inflammatory genes such as IL-2, IL-6, IL-8, IL-17A, and NF-κB. These alterations may be responsible for the observed organ injury and are directly proportional to the severity of the case. Additionally, they discovered that there was considerably low expression of two key genes (*PCDH9* and *NCAM*) in the tissues of all investigated organ types, which explained the low immune response and multi-organ failure seen in COVID-19 cases. Therefore, variations in the expression of many key genes were very helpful in the development of multiple therapeutic approaches. Furthermore, they investigated viral–host protein mapping and discovered that the interaction of viral proteins with host proteins may reduce the activity of eukaryotic initiation factor 2 (eIF2) and that eIF4-mediated host translation led to enhanced viral translation. Another transcriptomic analysis of patients with COVID-19 with mild, moderate, and severe outcomes found a differential gene expression signature between different outcomes that can be used to predict the clinical course of the disease and hence early intervention and management [44].

A study investigating viral gene expression patterns in mild and severe COVID-19 cases employed sc-RNAseq and bulk transcriptome analysis [58]. It revealed the presence of SARS-CoV-2 sequences in eight immune-related cell types, such as NK cells, macrophages, and T cells. The research showed that ORF10 was highly expressed in severe cases, while it was almost undetectable in mild cases. This led to a significant increase in the ORF10-to-nucleocapsid (N) expression ratio in severe cases. Furthermore, fatal cases exhibited distinct transcription regulatory sequences (TRSs) with a 5’ joint point at position 1073 of the SARS-CoV-2 genome, suggesting that these TRSs could act as predictive biomarkers [58]. These results indicate that some viral transcripts may be selectively expressed, potentially contributing to the virus’s increased virulence. If confirmed, this selective expression could be pivotal in understanding COVID-19’s pathophysiology and could be used to predict disease progression and mortality.

The ATGs and MAP1LC3C are two significant genes that determine the severity of COVID-19 cases and may be involved in disease progression. ATG genes’ translated protein is responsible for the production of autophagosomes and double-membrane vesicles [59,60,61]. Therefore, SARS-CoV-2 can depend on double-membrane vesicles for genome replication and protection from pathogen recognition receptors [62,63,64]. Conversely, a down-regulated complement system and autophagy genes may help slow the progression of the disease. Additionally, some patients with COVID-19 may benefit from the usage of autophagy inhibitors as an anti-inflammatory. The most well-known is azithromycin, a macrolide antibiotic that may significantly inhibit autophagosome function [65].

Notably, when evaluating the potential outcomes of SARS-CoV-2 infection, it is important to consider coinfection by opportunistic infections. Because opportunistic pathogens have been shown to upregulate complement-activating genes (C7, C8A, and C9) in mildly symptomatic patients, this raises the possibility that they are responsible for the severity of the cases [56,66]. Additionally, a substantially elevated ACE2 gene linked to interferon could potentially cause a severe inflammatory response in mildly symptomatic patients when compared to asymptomatic ones [67,68]. Other studies validated the finding of increased ACE2 expression in the nasopharyngeal tissue during transcriptome research [69]. Other genes were down-expressed in COVID-19 cases, such as transmembrane protease serine 2 (TMPRSS2); however, some investigations verified that there was no association between this gene expression and COVID-19 severity [70].

## 5. RNAseq Analysis in the Study of Long-Term Effects of COVID-19

A study on patients with COVID-19 at Jin Yintan Hospital in Wuhan, China, found that even two years after discharge, those with long-term symptoms experienced a lower quality of life, reduced physical ability, and increased mental health issues. These findings emphasize the lasting effects of COVID-19 on patients’ overall well-being [71]. This suggests that there is an urgent need for mechanistic knowledge of the long-term effects of COVID-19.

In this regard, several studies have analyzed different types of patient samples, including whole blood, blood components (such as plasma, monocytes, PBMCs, leukocytes, and T lymphocytes), nasal swabs, bronchoalveolar lavage, and tissue samples from various organs, including the heart, lungs, liver, and kidneys [72,73]. Based on the bulk and single-cell (sc) RNAseq data analysis, a recent study by Ilieva et al. found that patients with severe COVID-19 show changes in cell populations, particularly those of leukocytes and monocytes, which may result in cytokine storms and immune suppression [72]. Another study by Sommen et al. found that 13 genes were differentially expressed in the context of post-COVID-19 condition (PCC) and suggested that minor variations in the expression levels of genes related to innate immunity, such as higher expression of genes involved in interferon signaling, could indicate persistent, low-grade inflammation in young adults and adolescents affected by PCC [74]. Ryan et al. conducted a deep immunophenotyping analysis and found that recovering patients differed significantly from healthy controls in several innate immune cells (LD neutrophils, NK cells, and CXCR3+ monocytes) and adaptive immune cells (regulatory T cells, T helper, and T follicular helper) [75]. These differences were most pronounced at 12- and 16-weeks following infection.

RNA sequencing in COVID-19 survivors has shown notable changes in gene expression lasting up to six months after infection. These changes could affect recovery and responses to future infections and potentially worsen existing chronic conditions due to continued immune system activation. A study by Vaivode et al. used sc-RNAseq to explore immune dysregulation in patients with long COVID, identifying persistent immune cell changes months after infection [76]. These alterations, particularly in CD8+ T cells and neutrophils, suggest chronic immune activation contributing to long COVID symptoms. However, the limitations include the relatively small sample size and the lack of a clear understanding of the causal mechanisms behind the observed immune changes, which may affect the generalizability and long-term applicability of the findings. Additionally, the cross-sectional nature of the study did not allow for monitoring dynamic changes in the immune response over time [76].

Additionally, a study by Sommen et al. [74] investigated PCC in adolescents and young adults using RNAseq to analyze transcriptomic changes. The study found that specific gene expression patterns, particularly in immune response pathways, were significantly altered in individuals with PCC. Key pathways affected included those involved in inflammation, immune regulation, and cellular stress. The research highlighted differences in gene expression related to symptoms such as fatigue, cognitive dysfunction, and post-exertional malaise, providing insights into the biological underpinnings of long COVID in this age group. However, the study’s limitations include the relatively small sample size and its observational nature, which does not establish causality. Furthermore, the inclusion of participants without severe COVID-19 infections may limit the generalizability of the findings to critically ill populations [74].

Several studies have analyzed RNA sequencing and microarray data from patients with COVID-19, healthy individuals, and people with conditions like SARS, MERS, and lupus to investigate the biological mechanisms of SARS-CoV-2. Furthermore, they have advised conducting a more comprehensive analysis of RNAseq data linked to other OMICS data (such as proteomics, metabolomics, and genomics). These data should be thoroughly examined for gene regulatory networks using machine learning methods. Additionally, a thorough investigation of changes in gene expression may aid in the development of early diagnostic markers for long COVID [55,77,78].

## 6. RNAseq in Patients in the ICU with COVID-19 

Deep RNA sequencing (RNAseq) could play a valuable role in predicting ICU admission and mortality in critically ill patients with COVID-19 [43,79,80]. It might help greatly in determining pathogenic microorganisms (including SARS-CoV-2), gene expression as well as the host’s response. Sequencing SARS-CoV-2 samples might help substantially in understanding virus biology during active conditions. Secondary infections could also be detected and might have a great impact in determining the best-targeted antibiotics as well as immune-modulating targeted therapy. Moreover, significant differential expression in some genes such as PD-L1 and PD-L2, especially in critically ill patients with COVID-19. RNA sequencing analysis of patients in the ICU with COVID-19 showed significant differences in immune-regulating genes, such as PD-L1 and PD-L2, in those who died from the disease. Numerous other immune targets have been identified from these genomic changes. ITGB2 is an immune cell integrin that exhibits enhanced splicing of the last exon. Additionally, the authors discovered that the expression of the olfactory gene *OR6C4* was five times higher in patients who died. Furthermore, several proteins, such as HLA-C, HLA-E, NRP1, and NRP2 were affected by alternative transcription and splicing events. The entropy (>380,000 events) associated with principal component analysis (PCA) computed from alternative RNA splicing and transcription start/end can predict mortality in patients in the ICU with COVID-19 [43].

## 7. Aetiology of Variability of Host Responses on the Viral Pathogenesis in COVID-19

Patients with COVID-19 showed increased levels of infection-related pro-inflammatory cytokines, supporting the idea of a “cytokine storm”, a condition seen in SARS-CoV infections [81,82,83,84,85]. This mechanism leads to the recruitment and hyperactivation of inflammatory cells in the lungs, which in turn produces acute lung damage in the infected patients [86]. This highlights one potential molecular mechanism of COVID-19; however, other immune regulators, along with host genetic and epigenetic factors, could also play significant roles in shaping the disease’s manifestation [87,88,89,90]. In some CoV infections, host–pathogen interactions can have a double-edged effect since they may be advantageous to the viruses as well as the hosts [91]. A similar host–virus interaction may occur in COVID-19, potentially leading to more severe disease outcomes [92].

By studying the profile of both patients’ transcriptome in COVID-19 cases as well as virus genomic criteria isolated from the respective host, by taking nasopharyngeal samples. The researchers found characteristic and uncommon missense mutations in 3C-like protease in all patients under investigation. Further analyses showed that activation of innate immunity, interferon, and cytokine pathways might be responsible for the host responses. Moreover, in patients with COVID-19, there was a lack of apoptosis, phagosome, antigen presentation as well as hypoxia response. However, cytokine signaling genes, such as IL-1A, IL-6, TNF-α, IFN, CCL2, CCL4, CXCL2, and CCR1, were overexpressed, especially in the lung tissue. Meanwhile, the lung tissue lacked ACE2 upregulation; however, nasopharyngeal cells expressed high ACE2 and low DPP4. Remarkably, non-structural viral proteins exhibited the upregulation of integrins such as ITGAV, ITGA2B, ITGA4, ITGA5, ITGA6, ITGA8, ITGA9, ITGAE, ITGB3, and ITGB7 in the lungs compared to nasopharyngeal samples; these finding might raise the idea of the invasion potential way. Moreover, the upregulation of other lung transcription factors such as ATF3, GATA6, CBP, HDAC2, CEBP, NFAT, and TCF12 was demonstrated to possess a vital role in lung injury [69].

Recent findings indicate that ncRNAs and miRNAs may play a key role in the diverse ways hosts respond to COVID-19. For instance, miR-21-5p, which is elevated in severe cases, contributes to the inflammatory cytokine storm by promoting the production of cytokines and immune cell infiltration. This amplifies the inflammation observed in severe diseases, particularly in the lungs, contributing to the ARDS seen in critical cases. Research has demonstrated that miR-21-5p is crucial in regulating macrophage polarization, promoting a pro-inflammatory (M1) phenotype while inhibiting the anti-inflammatory (M2) phenotype, thereby intensifying inflammation [40,93,94].

By contrast, miR-146a, which typically acts as a negative regulator of immune responses, can help mitigate excessive inflammation. Dysregulation of miR-146a, often found in severe cases, may hinder this regulation, allowing uncontrolled inflammation, which contributes to tissue damage and severe outcomes of COVID-19 [39].

Additionally, miR-155, another key player in the immune response, is highly upregulated during COVID-19, especially in patients with comorbidities like chronic lung diseases. It has been linked to the development of hypercytokinemia (excessive cytokine production), a hallmark of severe disease. miR-155’s role in inflammatory responses and fibrosis makes it an important molecule in driving the pathology of severe COVID-19, particularly by inhibiting protective responses like endotoxin tolerance [38,95].

Another key miRNA, miR-451a, has been associated with inflammatory processes, and its downregulation in severe cases is linked to worsened outcomes. High levels of miR-451a correlate with an enhanced antiviral response and decreased lung injury, suggesting its potential as a therapeutic target [39,96]. Furthermore, miR-323-3p and miR-192-5p have been identified as prognostic biomarkers for severe outcomes, particularly in patients in the ICU. Low levels of these miRNAs have been associated with a higher likelihood of mortality [97].

Similarly, lncRNAs such as NEAT1 and MALAT1 have been linked to interferon regulation and immune cell activation, exacerbating inflammatory cascades during severe COVID-19 [98].

In summary, the diversity in host responses to SARS-CoV-2 is shaped by intricate immune processes, including heightened production of pro-inflammatory cytokines and the disrupted regulation of immune-related miRNAs and lncRNAs. These ncRNAs contribute to the severity of COVID-19 by modulating immune activation, inflammation, and viral clearance. Subsequently, RNAseq has emerged as a powerful tool in identifying these molecular signatures, aiding in the stratification of patients and the development of personalized therapeutic approaches.

## 8. Translating RNAseq Insights into Precision Medicine for COVID-19 Management

The integration of RNAseq findings into clinical workflows is paramount for bridging the gap between research and practical application. In precision medicine, RNAseq not only identifies molecular markers but also provides actionable insights that can inform patient management strategies, particularly in complex scenarios such as long COVID and critically ill patients in the ICU. Precision medicine aims to tailor treatment based on individual genetic, environmental, and lifestyle factors, and RNAseq serves as a cornerstone technology in this approach for managing patients with COVID-19 [99,100,101]. By identifying immune correlates of disease severity, RNAseq enables targeted therapeutic strategies. For instance, analysis of bronchoalveolar lavage samples has demonstrated that local immune patterns correlate with disease severity, aiding in tailored interventions [102].

Peripheral blood mononuclear cell (PBMC) analysis using RNAseq has revealed critical immune dynamics, such as increased circulating follicular helper T cells in mild cases and clonal expansion of cytotoxic CD8+ T cells in severe cases. Such findings highlight cellular targets for therapeutic interventions and patient stratification in ICU settings [103].

In addition, RNAseq rapidly identifies pathogens and their resistance profiles, expediting targeted antimicrobial therapy and reducing reliance on empirical treatments [43,104]. This application is particularly valuable for patients in the ICU for whom timely intervention is crucial.

Significant upregulation of immune checkpoint molecules, such as PD-1, and pro-inflammatory cytokines, including IL-2, IL-6, IL-8, IL-17A, and NF-κB, have been identified through RNAseq. These findings highlight the potential for immune-modulating therapies, particularly in ICU settings where cytokine storms pose a significant challenge [57,105,106,107]. Importantly, the variability in corticosteroid responses among patients underscores the need for precision approaches to immunomodulation [108,109,110].

Immune checkpoint inhibitors, such as anti-PD-L1 therapies, have shown promise in patients in the ICU with COVID-19, where upregulation of PD-L1 and PD-L2 has been linked to higher mortality [43,111,112,113]. By targeting these pathways, RNAseq-guided therapies could offer significant benefits.

Furthermore, RNAseq has identified viral genes, including the N protein and RNA-dependent RNA polymerase, as diagnostic and therapeutic targets [43,92]. Viral–host protein interaction mapping has also revealed mechanisms of viral manipulation of host translation, providing additional therapeutic avenues [57]

Moreover, RNAseq contributes to the understanding and management of long COVID by identifying persistent transcriptional signatures associated with immune dysregulation and chronic inflammation. This information can guide personalized rehabilitation strategies, addressing prolonged symptoms and improving patient outcomes [74,76].

Lastly, RNAseq’s ability to detect co-infections by identifying pathogen RNA enhances its utility in complex cases, particularly in ICU settings. This capability aligns with the broader goals of precision medicine by enabling accurate, comprehensive diagnoses to inform individualized treatment plans [42]. The Roles of RNAseq in the management of patients with COVID-19 are shown in Table 1.

**Table 1:** Roles of RNAseq in the management of patients with COVID-19.

Category	Key RNAseq Findings	Clinical Applications	References
Biomarkers of Disease Severity	Increased PD-1, IL-6, IL-8, TNF-α, NF-κB expression, alterations in immune cell populations (e.g., CD8+ T cells, follicular helper T cells) linked to severe disease.	− Identifying immune signatures for assessing disease severity and progression. − Predicting clinical outcomes based on cytokine expression profiles.	[43,57,103,105,106]
Therapeutic Targets	PD-1, PD-L1, IL-6, TNF-α, viral proteins like N protein, RNA-dependent RNA polymerase for antiviral therapy and immune checkpoint inhibition.	Identifying molecular targets for immune modulation and antiviral therapy.	[43,92,111,112]
Diagnosis of Co-infections	Identification of pathogen-specific RNA (e.g., bacterial species, fungal markers), co-infections detected by RNAseq.	Detecting bacterial, viral, or fungal co-infections alongside SARS-CoV-2.	[42]
Patient Stratification & Precision Therapy	Immune profiling (e.g., TCR/BCR analysis, cytokine profiling) for stratification of patients in the ICU, predicting corticosteroid efficacy, and tailoring immune-modulating therapies.	Stratifying patients for personalized therapies based on RNAseq profiles. Enhancing personalized treatment strategies through immune profiling and gene expression data.	[43,104]
Long COVID Management	Persistent immune activation, dysregulated cytokines, and T-cell exhaustion associated with long-term symptoms.	− Identifying persistent transcriptional signatures associated with immune dysregulation and chronic inflammation. − Personalized treatment strategies include immune modulation and cytokine-targeting therapies.	[74,76]

To translate RNAseq findings into clinical workflows, multidisciplinary approaches combining molecular biology, bioinformatics, and clinical expertise are essential. Developing user-friendly bioinformatics tools for real-time data interpretation and creating protocols for integrating RNAseq into electronic medical records would enhance its utility. Additionally, expanding collaborations between research institutions and healthcare providers will help establish standardized protocols for RNAseq applications, ensuring its broader adoption in clinical settings.

## 9. Barriers and Obstacles in Applying to Clinical Therapy

RNAseq has significantly enhanced our understanding of the molecular and cellular processes involved in COVID-19 pathology. However, its application in clinical areas like drug discovery still faces considerable challenges. Key obstacles include inconsistencies in sample collection, limited sample sizes, and variations in analytical methods, which limit the ability to accurately predict the effectiveness of experimental COVID-19 treatments [114].

Furthermore, ethical restrictions on patient sample collection and the limited availability of samples hinder more comprehensive studies, especially when it comes to analyzing tissues from different organs. Adherence to ethical guidelines such as the Declaration of Helsinki is critical. These guidelines mandate obtaining informed consent from all participants, ensuring they fully understand the purpose, procedures, risks, and potential benefits of the study. Ethical approval for such studies should be obtained through a recognized institutional review board (IRB) or ethics committee, which ensures that the research design complies with both national and international ethical standards [115].

Despite the potential of RNAseq to reveal vital biomarkers, most studies rely on samples like nasal swabs, bronchoalveolar lavage fluid, and PBMCs, often from critically ill patients, making it difficult to generalize findings across various stages of disease progression. Recent studies involving autopsy samples have provided significant insights into the biological impacts of SARS-CoV-2, particularly in the lungs, heart, kidneys, and liver [116]. However, these findings may not be representative of less severe or moderately ill patients due to the immunocompromised state of those from whom the autopsy samples are collected. Moreover, RNA-seq analysis from autopsy tissues often occurs after the disease has progressed significantly, which may alter cellular compositions and expression profiles, limiting their relevance to early-stage COVID-19 [117,118].

In addition to autopsy studies, the use of organoid models and animal studies is gaining traction as alternative methods to explore SARS-CoV-2 infection [116]. However, the limitations of organoid models, including the lack of immune systems and incomplete tissue mimicking, challenge the applicability of findings to human conditions. Animal models, such as rodents and non-human primates, have proven valuable for studying the immune response and pathophysiology of SARS-CoV-2, yet interspecies variations in susceptibility to the virus complicate the translation of findings to human cases [119]. As a result, there is a need to cross-validate data from organoid models with human samples to ensure clinical relevance.

The third approach involves using animal models to study SARS-CoV-2. This method allows researchers to control factors like virus dose, exposure time, and delivery. It also helps track tissue changes over time, from infection to recovery or death. Non-human primates, like macaques, are valuable due to their genetic similarity to humans, while rodents, such as hamsters and transgenic mice, are often used for their ease of breeding and low cost [120]. However, animals generally have lower infection rates compared to humans. Transcriptome analysis, combined with tracking TCR and BCR clonal expansion, helps identify immune factors that contribute to infection resistance. This can guide the development of more effective vaccines and therapeutic antibodies. For example, one study found transcriptomic patterns and TCR usage linked to a cytotoxic T-cell response to a specific epitope, which was associated with moderate illness and reduced viral replication in vitro. This epitope shows potential for future vaccine development. Another study, Scheid et al. identified potent virus-neutralizing antibodies from memory and activated B cells, which were linked to neutralizing activity in serum from patients who had recovered from COVID-19 [121]. scVDJ-seq, a technique derived from sc-RNAseq, facilitates the rapid identification of neutralizing antibodies against SARS-CoV-2, speeding up therapeutic antibody development. However, generating and testing thousands of recombinant antibodies to identify the most effective ones is time-consuming and costly. As such, skilled analysis of sc-RNAseq and scVDJ-seq data is necessary to efficiently select the best neutralizing antibodies [114].

Moreover, integrating ncRNAs and miRNAs into clinical practice poses challenges, despite their potential. Variability in RNAseq data due to patient heterogeneity, sampling methods, and disease stages complicates the identification of consistent ncRNA biomarkers [40]. Another challenge is the delivery of RNA-based therapies. miRNA and lncRNA-based therapeutics, including RNA mimics and inhibitors, hold promise for modulating immune responses and viral replication. However, these therapies face hurdles related to stability, specificity, and effective delivery to target tissues. Advances in nanotechnology and RNA delivery systems, including lipid nanoparticles and exosome-based platforms, are essential to overcoming these barriers [39,40].

Looking forward, the integration of emerging technologies such as long-read sequencing and single-cell RNAseq may overcome some of the current limitations of RNAseq in clinical applications. These advancements, combined with artificial intelligence (AI) and machine learning (ML), offer the potential for faster, more accurate diagnostic capabilities, which could significantly enhance patient stratification and therapeutic decision-making [52,122,123].

## 10. Conclusions

RNA sequencing has demonstrated clear advantages in understanding COVID-19’s molecular mechanisms, particularly in identifying blood transcriptome changes that reflect disease severity and host responses. This technique is pivotal in diagnosing and differentiating disease courses, especially for critically ill patients. By identifying key genes involved in inflammation and immune response, RNAseq facilitates precision medicine, helping clinicians select targeted therapies for patients, including those with severe COVID-19. The identification of novel therapeutic targets, such as inflammatory genes, RNA-dependent RNA polymerase, and immune checkpoint pathways, holds promise for future treatment strategies. Despite the progress, challenges remain in clinical translation due to ethical concerns, small sample sizes, and the gap between research and drug development. Future research should focus on overcoming these barriers to ensure that RNAseq’s potential is fully realized in clinical settings, improving patient outcomes and accelerating the development of tailored therapeutic approaches.

## References

[1] WHO (2018). Managing Epidemics: Key Facts About Major Deadly Diseases.

[2] Cen X., Wang F., Huang X., Jovic D., Dubee F., Yang H., Li Y. (2023). Towards precision medicine: Omics approach for COVID-19. Biosaf. Health.

[3] Abd El-Baky R.M., Shady E.R., Yahia R., Ahmed F.Y., Ramadan M., Ahmed H.R., Al-Kadmy I.M., Ramadan Y.N., Hetta H.F. (2023). COVID-19 associated Mucormycosis among ICU patients: Risk factors, control, and challenges. AMB Express.

[4] Abdelaal A., Abu-Elfatth A., Bakkar L.M., El-Azeem H.G.A., Hetta H.F., Badawy E.R. (2023). Assessment of COVID-19 associated coagulopathy and multiple hemostatic markers: A single center study in Egypt. Infection.

[5] Abd Ellah N.H., Gad S.F., Muhammad K., E Batiha G., Hetta H.F. (2020). Nanomedicine as a promising approach for diagnosis, treatment and prophylaxis against COVID-19. Nanomedicine.

[6] Brian D.A., Baric R.S. (2005). Coronavirus genome structure and replication. Curr. Top. Microbiol. Immunol..

[7] Gorbalenya A.E., Enjuanes L., Ziebuhr J., Snijder E.J. (2006). Nidovirales: Evolving the largest RNA virus genome. Virus Res..

[8] Waqar W., Ismail S., Jamil Z., Al-Shehhi A., Imran M., Hetta H. (2021). SARS-CoV-2 associated pathogenesis, immune dysfunction and involvement of host factors: A comprehensive review. Eur. Rev. Med. Pharmacol. Sci..

[9] Woo P.C., Lau S.K., Chu C.M., Chan K.H., Tsoi H.W., Huang Y., Wong B.H., Poon R.W., Cai J.J., Luk W.K. (2005). Characterization and complete genome sequence of a novel coronavirus, coronavirus HKU1, from patients with pneumonia. J. Virol..

[10] Zaki A.M., van Boheemen S., Bestebroer T.M., Osterhaus A.D., Fouchier R.A. (2012). Isolation of a novel coronavirus from a man with pneumonia in Saudi Arabia. N. Engl. J. Med..

[11] Wu F., Zhao S., Yu B., Chen Y.M., Wang W., Song Z.G., Hu Y., Tao Z.W., Tian J.H., Pei Y.Y. (2020). A new coronavirus associated with human respiratory disease in China. Nature.

[12] Shao N., Zhang C., Dong J., Sun L., Chen X., Xie Z., Xu B., An S., Zhang T., Yang F. (2022). Molecular evolution of human coronavirus-NL63, -229E, -HKU1 and -OC43 in hospitalized children in China. Front. Microbiol..

[13] Sayama Y., Okamoto M., Saito M., Saito-Obata M., Tamaki R., Joboco C.D., Lupisan S., Oshitani H. (2023). Seroprevalence of four endemic human coronaviruses and, reactivity and neutralization capability against SARS-CoV-2 among children in the Philippines. Sci. Rep..

[14] Belouzard S., Millet J.K., Licitra B.N., Whittaker G.R. (2012). Mechanisms of coronavirus cell entry mediated by the viral spike protein. Viruses.

[15] Dar H.A., Waheed Y., Najmi M.H., Ismail S., Hetta H.F., Ali A., Muhammad K. (2020). Multiepitope Subunit Vaccine Design against COVID-19 Based on the Spike Protein of SARS-CoV-2: An In Silico Analysis. J. Immunol. Res..

[16] Singhal T. (2020). A Review of Coronavirus Disease-2019 (COVID-19). Indian J. Pediatr..

[17] van der Hoek L. (2007). Human coronaviruses: What do they cause?. Antivir. Ther..

[18] Liu D.X., Liang J.Q., Fung T.S. (2021). Human Coronavirus-229E, -OC43, -NL63, and -HKU1 (Coronaviridae). Encycl. Virol..

[19] Mahmood Z., Alrefai H., Hetta H.F., Kader H.A., Munawar N., Abdul Rahman S., Elshaer S., Batiha G.E.-S., Muhammad K. (2020). Investigating virological, immunological, and pathological avenues to identify potential targets for developing covid-19 treatment and prevention strategies. Vaccines.

[20] Magdy Beshbishy A., Hetta H.F., Hussein D.E., Saati A.A., Uba C.C., Rivero-Perez N., Zaragoza-Bastida A., Shah M.A., Behl T., Batiha G.E.-S. (2020). Factors associated with increased morbidity and mortality of obese and overweight COVID-19 patients. Biology.

[21] Welch C. (2021). Age and frailty are independently associated with increased COVID-19 mortality and increased care needs in survivors: Results of an international multi-centre study. Age Ageing.

[22] Magdy Beshbishy A., Oti V.B., Hussein D.E., Rehan I.F., Adeyemi O.S., Rivero-Perez N., Zaragoza-Bastida A., Shah M.A., Abouelezz K., Hetta H.F. (2021). Factors behind the higher COVID-19 risk in diabetes: A critical review. Front. Public Health.

[23] Ksiazek T.G., Erdman D., Goldsmith C.S., Zaki S.R., Peret T., Emery S., Tong S., Urbani C., Comer J.A., Lim W. (2003). A novel coronavirus associated with severe acute respiratory syndrome. N. Engl. J. Med..

[24] Hajjar S.A., Memish Z.A., McIntosh K. (2013). Middle East Respiratory Syndrome Coronavirus (MERS-CoV): A perpetual challenge. Ann. Saudi Med..

[25] Batiha G.E.-S., Zayed M.A., Awad A.A., Shaheen H.M., Mustapha S., Herrera-Calderon O., Pagnossa J.P., Algammal A.M., Zahoor M., Adhikari A. (2021). Management of SARS-CoV-2 infection: Key focus in macrolides efficacy for COVID-19. Front. Med..

[26] Sohrabi C., Alsafi Z., O’Neill N., Khan M., Kerwan A., Al-Jabir A., Iosifidis C., Agha R. (2020). World Health Organization declares global emergency: A review of the 2019 novel coronavirus (COVID-19). Int. J. Surg..

[27] WHO Number of COVID-19 Cases Reported to WHO. https://data.who.int/dashboards/covid19/cases?n=c.

[28] Lu R., Zhao X., Li J., Niu P., Yang B., Wu H., Wang W., Song H., Huang B., Zhu N. (2020). Genomic characterisation and epidemiology of 2019 novel coronavirus: Implications for virus origins and receptor binding. Lancet.

[29] Wan Y., Shang J., Graham R., Baric R.S., Li F. (2020). Receptor recognition by the novel coronavirus from Wuhan: An analysis based on decade-long structural studies of SARS coronavirus. J. Virol..

[30] Hetta H., Muhammad K., Algammal A., Ramadan H., Abdel-Rahman M., Mabrok M., Koneru G., Elkady A., Batiha G., Waheed Y. (2021). Mapping the effect of drugs on ACE2 as a novel target site for COVID-19 therapy. Eur. Rev. Med. Pharmacol. Sci..

[31] Pal P.K., Chattopadhyay A., Bandyopadhyay D. (2020). Melatonin as a potential therapeutic molecule against COVID-19 associated gastrointestinal complications: An unrevealed link. Melatonin Res..

[32] COVIDSurg C., GlobalSurg C. (2022). SARS-CoV-2 infection and venous thromboembolism after surgery: An international prospective cohort study. Anaesthesia.

[33] Glasbey J., Ademuyiwa A., Adisa A., AlAmeer E., Arnaud A.P., Ayasra F., Azevedo J., Minaya-Bravo A., Costas-Chavarri A., Edwards J. (2021). Effect of COVID-19 pandemic lockdowns on planned cancer surgery for 15 tumour types in 61 countries: An international, prospective, cohort study. Lancet Oncol..

[34] Batiha G.E.-S., Moubarak M., Shaheen H.M., Zakariya A.M., Usman I.M., Rauf A., Adhikari A., Dey A., Alexiou A., Hetta H.F. (2022). Favipiravir in SARS-CoV-2 infection: Is it worth it?. Comb. Chem. High Throughput Screen..

[35] Farghly Youssif S., Abdelrady M.M., Thabet A.A., Abdelhamed M.A., Gad M.O.A., Abu-Elfatth A.M., Saied G.M., Goda I., Algammal A.M., Batiha G.E.-S. (2022). COVID-19 associated mucormycosis in Assiut University Hospitals: A multidisciplinary dilemma. Sci. Rep..

[36] Moubarak M., Kasozi K.I., Hetta H.F., Shaheen H.M., Rauf A., Al-Kuraishy H.M., Qusti S., Alshammari E.M., Ayikobua E.T., Ssempijja F. (2021). The rise of SARS-CoV-2 variants and the role of convalescent plasma therapy for management of infections. Life.

[37] Ramadan Y.N., Kamel A.M., Medhat M.A., Hetta H.F. (2024). MicroRNA signatures in the pathogenesis and therapy of inflammatory bowel disease. Clin. Exp. Med..

[38] Farr R.J., Rootes C.L., Rowntree L.C., Nguyen T.H.O., Hensen L., Kedzierski L., Cheng A.C., Kedzierska K., Au G.G., Marsh G.A. (2021). Altered microRNA expression in COVID-19 patients enables identification of SARS-CoV-2 infection. PLoS Pathog..

[39] Lin Y., Sun Q., Zhang B., Zhao W., Shen C. (2023). The regulation of lncRNAs and miRNAs in SARS-CoV-2 infection. Front. Cell Dev. Biol..

[40] Ayoub S.E., Shaker O.G., Masoud M., Hassan E.A., Ezzat E.M., Ahmed M.I., Ahmed R.I., Amin A.A.I., Abd El Reheem F., Khalefa A.A. (2024). Altered expression of serum lncRNA CASC2 and miRNA-21-5p in COVID-19 patients. Hum. Genom..

[41] Mimmi S., Zimbo A.M., Rotundo S., Cione E., Nisticò N., Aloisio A., Maisano D., Tolomeo A.M., Dattilo V., Lionello R. (2023). SARS CoV-2 spike protein-guided exosome isolation facilitates detection of potential miRNA biomarkers in COVID-19 infections. Clin. Chem. Lab. Med..

[42] Wargodsky R., Dela Cruz P., LaFleur J., Yamane D., Kim J.S., Benjenk I., Heinz E., Irondi O.O., Farrar K., Toma I. (2022). RNA Sequencing in COVID-19 patients identifies neutrophil activation biomarkers as a promising diagnostic platform for infections. PLoS ONE.

[43] Fredericks A.M., Jentzsch M.S., Cioffi W.G., Cohen M., Fairbrother W.G., Gandhi S.J., Harrington E.O., Nau G.J., Reichner J.S., Ventetuolo C.E. (2022). Deep RNA sequencing of intensive care unit patients with COVID-19. Sci. Rep..

[44] Jain R., Ramaswamy S., Harilal D., Uddin M., Loney T., Nowotny N., Alsuwaidi H., Varghese R., Deesi Z., Alkhajeh A. (2021). Host transcriptomic profiling of COVID-19 patients with mild, moderate, and severe clinical outcomes. Comput. Struct. Biotechnol. J..

[45] Hadzega D., Babisova K., Hyblova M., Janostiakova N., Sabaka P., Janega P., Minarik G. (2024). Analysis of transcriptomics data from COVID-19 patients: A pilot research. Folia Microbiol..

[46] Oliveira T.T., Freitas J.F., de Medeiros V.P.B., Xavier T.J.d.S., Agnez-Lima L.F. (2024). Integrated analysis of RNA-seq datasets reveals novel targets and regulators of COVID-19 severity. Life Sci. Alliance.

[47] Yang J., Yan Y., Zhong W. (2021). Application of omics technology to combat the COVID-19 pandemic. MedComm.

[48] Samy A., Maher M.A., Abdelsalam N.A., Badr E. (2022). SARS-CoV-2 potential drugs, drug targets, and biomarkers: A viral-host interaction network-based analysis. Sci. Rep..

[49] Daamen A.R., Bachali P., Bonham C.A., Somerville L., Sturek J.M., Grammer A.C., Kadl A., Lipsky P.E. (2022). COVID-19 patients exhibit unique transcriptional signatures indicative of disease severity. Front. Immunol..

[50] Kwan P.K.W., Cross G.B., Naftalin C.M., Ahidjo B.A., Mok C.K., Fanusi F., Permata Sari I., Chia S.C., Kumar S.K., Alagha R. (2021). A blood RNA transcriptome signature for COVID-19. BMC Med. Genom..

[51] Rodriguez C., de Prost N., Fourati S., Lamoureux C., Gricourt G., N’debi M., Canoui-Poitrine F., Désveaux I., Picard O., Demontant V. (2021). Viral genomic, metagenomic and human transcriptomic characterization and prediction of the clinical forms of COVID-19. PLoS Pathog..

[52] Iqbal N., Kumar P. (2022). Integrated COVID-19 Predictor: Differential expression analysis to reveal potential biomarkers and prediction of coronavirus using RNA-Seq profile data. Comput. Biol. Med..

[53] Abdellatif A.A., Tawfeek H.M., Abdelfattah A., Batiha G.E.-S., Hetta H.F. (2021). Recent updates in COVID-19 with emphasis on inhalation therapeutics: Nanostructured and targeting systems. J. Drug Deliv. Sci. Technol..

[54] Abid S.A., Muneer A.A., Al-Kadmy I.M., Sattar A.A., Beshbishy A.M., Batiha G.E.-S., Hetta H.F. (2021). Biosensors as a future diagnostic approach for COVID-19. Life Sci..

[55] Cavalli E., Petralia M.C., Basile M.S., Bramanti A., Bramanti P., Nicoletti F., Spandidos D.A., Shoenfeld Y., Fagone P. (2020). Transcriptomic analysis of COVID-19 lungs and bronchoalveolar lavage fluid samples reveals predominant B cell activation responses to infection. Int. J. Mol. Med..

[56] Faridl M., Mellyani K., Khoirunnisa K., Septiani P., Giri-Rachman E.A., Nugrahapraja H., Rahmawati E., Alamanda C.N.C., Ristandi R.B., Rachman R.W. (2022). RNA sequence analysis of nasopharyngeal swabs from asymptomatic and mildly symptomatic patients with COVID-19. Int. J. Infect. Dis. IJID Off. Publ. Int. Soc. Infect. Dis..

[57] Bass A., Liu Y., Dakshanamurthy S. (2021). Single-Cell and Bulk RNASeq Profiling of COVID-19 Patients Reveal Immune and Inflammatory Mechanisms of Infection-Induced Organ Damage. Viruses.

[58] Liu T., Jia P., Fang B., Zhao Z. (2020). Differential Expression of Viral Transcripts From Single-Cell RNA Sequencing of Moderate and Severe COVID-19 Patients and Its Implications for Case Severity. Front. Microbiol..

[59] Andaloussi A.E., Habib S., Soylemes G., Laknaur A., Elhusseini H., Al-Hendy A., Ismail N. (2017). Defective expression of ATG4D abrogates autophagy and promotes growth in human uterine fibroids. Cell Death Discov..

[60] Bonam S.R., Bayry J., Tschan M.P., Muller S. (2020). Progress and Challenges in The Use of MAP1LC3 as a Legitimate Marker for Measuring Dynamic Autophagy In Vivo. Cells.

[61] Yun E.-J., Kim S., Hsieh J.-T., Baek S.T. (2020). Wnt/β-catenin signaling pathway induces autophagy-mediated temozolomide-resistance in human glioblastoma. Cell Death Dis..

[62] Shroff A., Nazarko T.Y. (2021). The Molecular Interplay between Human Coronaviruses and Autophagy. Cells.

[63] Wolff G., Limpens R., Zevenhoven-Dobbe J.C., Laugks U., Zheng S., de Jong A.W.M., Koning R.I., Agard D.A., Grünewald K., Koster A.J. (2020). A molecular pore spans the double membrane of the coronavirus replication organelle. Science.

[64] Wolff G., Melia C.E., Snijder E.J., Bárcena M. (2020). Double-Membrane Vesicles as Platforms for Viral Replication. Trends Microbiol..

[65] Venditto V.J., Haydar D., Abdel-Latif A., Gensel J.C., Anstead M.I., Pitts M.G., Creameans J., Kopper T.J., Peng C., Feola D.J. (2021). Immunomodulatory Effects of Azithromycin Revisited: Potential Applications to COVID-19. Front. Immunol..

[66] Li Q., Chen Z. (2021). An update: The emerging evidence of complement involvement in COVID-19. Med. Microbiol. Immunol..

[67] Ziegler C.G.K., Allon S.J., Nyquist S.K., Mbano I.M., Miao V.N., Tzouanas C.N., Cao Y., Yousif A.S., Bals J., Hauser B.M. (2020). SARS-CoV-2 Receptor ACE2 Is an Interferon-Stimulated Gene in Human Airway Epithelial Cells and Is Detected in Specific Cell Subsets across Tissues. Cell.

[68] Ramasamy S., Subbian S. (2021). Critical Determinants of Cytokine Storm and Type I Interferon Response in COVID-19 Pathogenesis. Clin. Microbiol. Rev..

[69] Islam A.B.M.M.K., Khan M.A.-A.-K., Ahmed R., Hossain M.S., Kabir S.M.T., Islam M.S., Siddiki A.M.A.M.Z. (2021). Transcriptome of nasopharyngeal samples from COVID-19 patients and a comparative analysis with other SARS-CoV-2 infection models reveal disparate host responses against SARS-CoV-2. J. Transl. Med..

[70] Rossi Á.D., de Araújo J.L.F., de Almeida T.B., Ribeiro-Alves M., de Almeida Velozo C., Almeida J.M., de Carvalho Leitão I., Ferreira S.N., da Silva Oliveira J., Alves H.J. (2021). Association between ACE2 and TMPRSS2 nasopharyngeal expression and COVID-19 respiratory distress. Sci. Rep..

[71] Huang L., Li X., Gu X., Zhang H., Ren L., Guo L., Liu M., Wang Y., Cui D., Wang Y. (2022). Health outcomes in people 2 years after surviving hospitalisation with COVID-19: A longitudinal cohort study. Lancet. Respir. Med..

[72] Ilieva M., Tschaikowski M., Vandin A., Uchida S. (2022). The current status of gene expression profilings in COVID-19 patients. Clin. Transl. Discov..

[73] Ghandikota S., Sharma M., Jegga A.G. (2021). Computational workflow for functional characterization of COVID-19 through secondary data analysis. STAR Protoc..

[74] Sommen S.L., Zhao Z., Segtnan S., Stiansen-Sonerud T., Selvakumar J., Beier Havdal L., Gjerstad J., Wyller V.B.B., Lund Berven L. (2024). Bulk RNA sequencing for analysis of post COVID-19 condition in adolescents and young adults. J. Transl. Med..

[75] Ryan F.J., Hope C.M., Masavuli M.G., Lynn M.A., Mekonnen Z.A., Yeow A.E.L., Garcia-Valtanen P., Al-Delfi Z., Gummow J., Ferguson C. (2022). Long-term perturbation of the peripheral immune system months after SARS-CoV-2 infection. BMC Med..

[76] Vaivode K., Saksis R., Litvina H.D., Niedra H., Spriņģe M.L., Krūmiņa U., Kloviņš J., Rovite V. (2024). Single-Cell RNA Sequencing Reveals Alterations in Patient Immune Cells with Pulmonary Long COVID-19 Complications. Curr. Issues Mol. Biol..

[77] Cao Y., Xu X., Kitanovski S., Song L., Wang J., Hao P., Hoffmann D. (2021). Comprehensive Comparison of RNA-Seq Data of SARS-CoV-2, SARS-CoV and MERS-CoV Infections: Alternative Entry Routes and Innate Immune Responses. Front. Immunol..

[78] Jha P.K., Vijay A., Halu A., Uchida S., Aikawa M. (2020). Gene Expression Profiling Reveals the Shared and Distinct Transcriptional Signatures in Human Lung Epithelial Cells Infected with SARS-CoV-2, MERS-CoV, or SARS-CoV: Potential Implications in Cardiovascular Complications of COVID-19. Front. Cardiovasc. Med..

[79] Penrice-Randal R., Dong X., Shapanis A.G., Gardner A., Harding N., Legebeke J., Lord J., Vallejo A.F., Poole S., Brendish N.J. (2022). Blood gene expression predicts intensive care unit admission in hospitalised patients with COVID-19. Front. Immunol..

[80] López-Martínez C., Martín-Vicente P., Gómez de Oña J., López-Alonso I., Gil-Peña H., Cuesta-Llavona E., Fernández-Rodríguez M., Crespo I., Salgado del Riego E., Rodríguez-García R. (2023). Transcriptomic clustering of critically ill COVID-19 patients. Eur. Respir. J..

[81] Mehta P., McAuley D.F., Brown M., Sanchez E., Tattersall R.S., Manson J.J. (2020). COVID-19: Consider cytokine storm syndromes and immunosuppression. Lancet.

[82] Khalaf M., Alboraie M., Abdel-Gawad M., Abdelmalek M., Abu-Elfatth A., Abdelhamed W., Zaghloul M., ElDeeb R., Abdeltwab D., Abdelghani M. (2022). Prevalence and predictors of persistent symptoms after clearance of SARS-CoV-2 infection: A multicenter study from Egypt. Infect. Drug Resist..

[83] Collaborative G., Collaborative C. (2021). SARS-CoV-2 vaccination modelling for safe surgery to save lives: Data from an international prospective cohort study. Br. J. Surg..

[84] Batiha G.E.-S., Alqarni M., Awad D.A., Algammal A.M., Nyamota R., Wahed M.I., Shah M.A., Amin M.N., Adetuyi B.O., Hetta H.F. (2021). Dairy-derived and egg white proteins in enhancing immune system against COVID-19. Front. Nutr..

[85] Kasozi K.I., Niedbała G., Alqarni M., Zirintunda G., Ssempijja F., Musinguzi S.P., Usman I.M., Matama K., Hetta H.F., Mbiydzenyuy N.E. (2020). Bee venom—A potential complementary medicine candidate for SARS-CoV-2 infections. Front. Public Health.

[86] Ye Q., Wang B., Mao J. (2020). The pathogenesis and treatment of the ‘Cytokine Storm’ in COVID-19. J. Infect.

[87] Schäfer A., Baric R.S. (2017). Epigenetic Landscape during Coronavirus Infection. Pathogens.

[88] Gu J., Korteweg C. (2007). Pathology and pathogenesis of severe acute respiratory syndrome. Am. J. Pathol..

[89] Kaidashev I., Shlykova O., Izmailova O., Torubara O., Yushchenko Y., Tyshkovska T., Kyslyi V., Belyaeva A., Maryniak D. (2021). Host gene variability and SARS-CoV-2 infection: A review article. Heliyon.

[90] Altiok D., Savci E.Z., Özkara B., Alkan K., Namdar D.S., Tunçer G., Kilinç B.R., Suiçmez E., Çetin G., Ünal S. (2021). Host variations in SARS-CoV-2 infection. Turk. J. Biol..

[91] Fung T.S., Liu D.X. (2019). Human Coronavirus: Host-Pathogen Interaction. Annu. Rev. Microbiol..

[92] Gordon D.E., Jang G.M., Bouhaddou M., Xu J., Obernier K., White K.M., O’Meara M.J., Rezelj V.V., Guo J.Z., Swaney D.L. (2020). A SARS-CoV-2 protein interaction map reveals targets for drug repurposing. Nature.

[93] Farr R.J., Rootes C.L., Stenos J., Foo C.H., Cowled C., Stewart C.R. (2022). Detection of SARS-CoV-2 infection by microRNA profiling of the upper respiratory tract. PLoS ONE.

[94] Wang Z., Brandt S., Medeiros A., Wang S., Wu H., Dent A., Serezani C.H. (2015). MicroRNA 21 Is a Homeostatic Regulator of Macrophage Polarization and Prevents Prostaglandin E2-Mediated M2 Generation. PLoS ONE.

[95] Kruglikov I.L., Scherer P.E. (2021). Preexisting and inducible endotoxemia as crucial contributors to the severity of COVID-19 outcomes. PLoS Pathog..

[96] Wilson J.C., Kealy D., James S.R., Plowman T., Newling K., Jagger C., Filbey K., Mann E.R., Konkel J.E., Menon M. (2022). Integrated miRNA/cytokine/chemokine profiling reveals severity-associated step changes and principal correlates of fatality in COVID-19. iScience.

[97] Smail S.W., Hirmiz S.M., Ahmed A.A., Albarzinji N., Awla H.K., Amin K., Janson C. (2024). Decoding the intricacies: A comprehensive analysis of microRNAs in the pathogenesis, diagnosis, prognosis and therapeutic strategies for COVID-19. Front. Med..

[98] Huang K., Wang C., Vagts C., Raguveer V., Finn P.W., Perkins D.L. (2022). Long non-coding RNAs (lncRNAs) NEAT1 and MALAT1 are differentially expressed in severe COVID-19 patients: An integrated single-cell analysis. PLoS ONE.

[99] Manzari M.T., Shamay Y., Kiguchi H., Rosen N., Scaltriti M., Heller D.A. (2021). Targeted drug delivery strategies for precision medicines. Nat. Rev. Mater..

[100] Arriaga-Canon C., Contreras-Espinosa L., Rebollar-Vega R., Montiel-Manríquez R., Cedro-Tanda A., García-Gordillo J.A., Álvarez-Gómez R.M., Jiménez-Trejo F., Castro-Hernández C., Herrera L.A. (2022). Transcriptomics and RNA-Based Therapeutics as Potential Approaches to Manage SARS-CoV-2 Infection. Int. J. Mol. Sci..

[101] Van de Sande B., Lee J.S., Mutasa-Gottgens E., Naughton B., Bacon W., Manning J., Wang Y., Pollard J., Mendez M., Hill J. (2023). Applications of single-cell RNA sequencing in drug discovery and development. Nat. Rev. Drug Discov..

[102] Wauters E., Van Mol P., Garg A.D., Jansen S., Van Herck Y., Vanderbeke L., Bassez A., Boeckx B., Malengier-Devlies B., Timmerman A. (2021). Discriminating mild from critical COVID-19 by innate and adaptive immune single-cell profiling of bronchoalveolar lavages. Cell Res..

[103] Stephenson E., Reynolds G., Botting R.A., Calero-Nieto F.J., Morgan M.D., Tuong Z.K., Bach K., Sungnak W., Worlock K.B., Yoshida M. (2021). Single-cell multi-omics analysis of the immune response in COVID-19. Nat. Med..

[104] Feng Y., Ling Y., Bai T., Xie Y., Huang J., Li J., Xiong W., Yang D., Chen R., Lu F. (2020). COVID-19 with Different Severities: A Multicenter Study of Clinical Features. Am. J. Respir. Crit. Care Med..

[105] Bouadma L., Wiedemann A., Patrier J., Surénaud M., Wicky P.H., Foucat E., Diehl J.L., Hejblum B.P., Sinnah F., de Montmollin E. (2020). Immune Alterations in a Patient with SARS-CoV-2-Related Acute Respiratory Distress Syndrome. J. Clin. Immunol..

[106] Bellesi S., Metafuni E., Hohaus S., Maiolo E., Marchionni F., D’Innocenzo S., La Sorda M., Ferraironi M., Ramundo F., Fantoni M. (2020). Increased CD95 (Fas) and PD-1 expression in peripheral blood T lymphocytes in COVID-19 patients. Br. J. Haematol..

[107] Vlasova-St. Louis I., Fang D., Amer Y., Mohei H. (2023). COVID-19-Omics Report: From Individual Omics Approaches to Precision Medicine. Reports.

[108] Horby P., Lim W.S., Emberson J.R., Mafham M., Bell J.L., Linsell L., Staplin N., Brightling C., Ustianowski A., Elmahi E. (2021). Dexamethasone in Hospitalized Patients with COVID-19. N. Engl. J. Med..

[109] Prescott H.C., Rice T.W. (2020). Corticosteroids in COVID-19 ARDS: Evidence and Hope During the Pandemic. JAMA.

[110] Waterer G.W., Rello J. (2020). Steroids and COVID-19: We Need a Precision Approach, Not One Size Fits All. Infect. Dis. Ther..

[111] Chavez-Galan L., Ruiz A., Martinez-Espinosa K., Aguilar-Duran H., Torres M., Falfan-Valencia R., Pérez-Rubio G., Selman M., Buendia-Roldan I. (2022). Circulating Levels of PD-L1, TIM-3 and MMP-7 Are Promising Biomarkers to Differentiate COVID-19 Patients That Require Invasive Mechanical Ventilation. Biomolecules.

[112] Pezeshki P.S., Rezaei N. (2021). Immune checkpoint inhibition in COVID-19: Risks and benefits. Expert Opin. Biol. Ther..

[113] Vivarelli S., Falzone L., Grillo C.M., Scandurra G., Torino F., Libra M. (2020). Cancer Management during COVID-19 Pandemic: Is Immune Checkpoint Inhibitors-Based Immunotherapy Harmful or Beneficial?. Cancers.

[114] Liu W., Ye X., An Z., Zhao Z. (2022). The challenges and opportunities of scRNA-seq in COVID-19 research and clinical translation. Virol. J..

[115] Goodyear M.D., Krleza-Jeric K., Lemmens T. (2007). The Declaration of Helsinki. BMJ (Clin. Res. Ed.).

[116] Tran B.M., Deliyannis G., Hachani A., Earnest L., Torresi J., Vincan E. (2022). Organoid Models of SARS-CoV-2 Infection: What Have We Learned about COVID-19?. Organoids.

[117] Delorey T.M., Ziegler C.G., Heimberg G., Normand R., Yang Y., Segerstolpe Å., Abbondanza D., Fleming S.J., Subramanian A., Montoro D.T. (2021). COVID-19 tissue atlases reveal SARS-CoV-2 pathology and cellular targets. Nature.

[118] Melms J.C., Biermann J., Huang H., Wang Y., Nair A., Tagore S., Katsyv I., Rendeiro A.F., Amin A.D., Schapiro D. (2021). A molecular single-cell lung atlas of lethal COVID-19. Nature.

[119] Jansen J., Reimer K.C., Nagai J.S., Varghese F.S., Overheul G.J., de Beer M., Roverts R., Daviran D., Fermin L.A., Willemsen B. (2022). SARS-CoV-2 infects the human kidney and drives fibrosis in kidney organoids. Cell Stem Cell.

[120] Speranza E., Williamson B.N., Feldmann F., Sturdevant G.L., Pérez-Pérez L., Meade-White K., Smith B.J., Lovaglio J., Martens C., Munster V.J. (2021). Single-cell RNA sequencing reveals SARS-CoV-2 infection dynamics in lungs of African green monkeys. Sci. Transl. Med..

[121] Scheid J.F., Barnes C.O., Eraslan B., Hudak A., Keeffe J.R., Cosimi L.A., Brown E.M., Muecksch F., Weisblum Y., Zhang S. (2021). B cell genomics behind cross-neutralization of SARS-CoV-2 variants and SARS-CoV. Cell.

[122] Liu X., Shi J., Jiao Y., An J., Tian J., Yang Y., Zhuo L. (2024). Integrated multi-omics with machine learning to uncover the intricacies of kidney disease. Brief. Bioinform..

[123] Sameh M., Khalaf H.M., Anwar A.M., Osama A., Ahmed E.A., Mahgoub S., Ezzeldin S., Tanios A., Alfishawy M., Said A.F. (2023). Integrated multiomics analysis to infer COVID-19 biological insights. Sci. Rep..

